# MALDI-TOF Mass Spectrometry for Multilocus Sequence Typing of *Escherichia coli* Reveals Diversity among Isolates Carrying *bla*_CMY-2_-Like Genes

**DOI:** 10.1371/journal.pone.0143446

**Published:** 2015-11-20

**Authors:** Kaitlin A. Tagg, Andrew N. Ginn, Sally R. Partridge, Jonathan R. Iredell

**Affiliations:** Centre for Infectious Diseases and Microbiology, The Westmead Institute for Medical Research, The University of Sydney and Westmead Hospital, Westmead, New South Wales, Australia; Catalan Institute for Water Research (ICRA), SPAIN

## Abstract

Effective surveillance and management of pathogenic *Escherichia coli* relies on robust and reproducible typing methods such as multilocus sequence typing (MLST). Typing of *E*. *coli* by MLST enables tracking of pathogenic clones that are known to carry virulence factors or spread resistance, such as the globally-prevalent ST131 lineage. Standard MLST for *E*. *coli* requires sequencing of seven alleles, or a whole genome, and can take several days. Here, we have developed and validated a nucleic-acid-based MALDI-TOF mass spectrometry (MS) method for MLST as a rapid alternative to sequencing that requires minimal operator expertise. Identification of alleles was 99.6% concordant with sequencing. We employed MLST by MALDI-TOF MS to investigate diversity among 62 *E*. *coli* isolates from Sydney, Australia, carrying a *bla*_CMY-2_-like gene on an IncI1 plasmid to determine whether any dominant clonal lineages are associated with the spread of this globally-disseminated resistance gene. Thirty-four known sequence types were identified, including lineages associated with human disease, animal and environmental sources. This suggests that the dissemination of *bla*_CMY-2_-like-genes is more complex than the simple spread of successful pathogenic clones. *E*. *coli* MLST by MALDI-TOF MS, employed here for the first time, can be utilised as an automated tool for large-scale population analyses or for targeted screening for known high-risk clones in a diagnostic setting.

## Introduction

*Escherichia coli* is an important commensal and pathogenic organism and a leading cause of urinary tract infections, diarrhoeal diseases and bacteraemia worldwide [1]. Effective infection control and management of these organisms requires reliable and robust subtyping schemes. Fine-scale pulsed-field gel electrophoresis (PFGE) is considered the ‘gold standard’ for tracking local outbreaks, as it has the power to detect variation between closely related organisms [2]. Typing based on multiple-locus variable number tandem repeat analysis (MLVA) [3, 4] is more rapid and reproducible than PFGE and is increasingly being adopted [5]. However, surveillance for organisms of particular lineages that may not necessarily be part of a specific outbreak requires typing schemes that can elucidate more distant evolutionary relationships such as multilocus sequence typing (MLST).

MLST utilises genetic polymorphisms within well-conserved chromosomal ‘housekeeping’ genes to define the general relatedness of bacterial strains. Each of these loci (seven for *E*. *coli*) may have several hundred variants that are given different allele numbers. The combination of all seven allele numbers defines the sequence type (ST) of a strain [6]. Reported STs are collated in an online MLST database (http://mlst.warwick.ac.uk/mlst/dbs/Ecoli is the most widely used for *E*. *coli*) that is updated regularly to include new allelic variants and STs. STs can be further grouped into ST complexes (STC) (clonal complexes) that contain STs that vary at only one or two alleles and are therefore deemed to be related lineages. Typing by MLST has enabled the recognition of important *E*. *coli* strain types (‘clones’) that are more virulent or likely to spread resistance, including the globally-successful ST131 clonal lineage [7, 8].

MLST by traditional Sanger sequencing is simple and highly reproducible but is relatively labour-intensive for larger sets of isolates. Whole-genome sequencing (WGS) allows extraction of MLST data with relative ease but sample preparation time and processing procedures mean that results are often not available for several weeks. Nucleic-acid-based MALDI-TOF mass spectrometry (MALDI-TOF MS) is a rapid method that has been successfully employed for MLST of bacterial pathogens, including *Neisseria meningitidis* and *Streptococcus pneumoniae* [9–11]. This method involves initial PCR amplification followed by *in vitro* transcription and base-specific cleavage to generate fragments small enough for detection by MS, which are then compared to a reference library, similar to protein-based MALDI-TOF MS methods [12].

Nucleic-acid-based MALDI-TOF MS is increasingly being utilised for characterisation of pathogenic organisms including single nucleotide polymorphism (SNP) detection in *Staphylococcus aureus* [13], identification of human papilloma virus subtypes [14] and monitoring of hepatitis B virus quasispecies during treatment [15]. The high level of automation ensures reproducibility and minimises costs as well as reducing the need for specialist technical expertise. The rapid availability of MALDI-TOF MS results can inform short-term infection control strategies and over time will enable the accumulation of valuable epidemiological information, both of which are important for improving public health outcomes.

Here we describe the development and validation of a MALDI-TOF MS method for MLST of *E*. *coli*. We employed this method to investigate clonal diversity among *E*. *coli* strains from Sydney, Australia, carrying the plasmid-borne AmpC β-lactamase (pAmpC) gene *bla*_CMY-2_ and variants [16]. *bla*_CMY-2_ is the most common pAmpC gene worldwide, particularly in *E*. *coli*, and although found on several distinct plasmid lineages (IncI1, F, A/C, K and B/O) [17–19], it is predominantly associated with IncI1 plasmids in Australia [20, 21].

## Materials and Methods

### Bacterial isolates

A set of 33 *E*. *coli* isolates with STs determined from Illumina MiSeq sequences using the MLST finder database (https://cge.cbs.dtu.dk/services/MLST), or by conventional Sanger sequencing (http://mlst.warwick.ac.uk/mlst/dbs/Ecoli) [22, 23], was used for validation of MLST by MALDI-TOF MS and included 10 examples of ST131 and representatives of 14 other STs. Crude bacterial lysates, prepared by suspending a loopful of colonies in 1 ml sterile distilled water and boiling for 10–20 min, were used as templates for all PCRs. Seventy-two *E*. *coli* isolates identified as carrying both a *bla*_CMY-2_-like gene and an IncI1 plasmid in previous surveys (Feb 2005-April 2014 [21, 24]) were investigated to identify those in which the *bla*_CMY-2_-like gene is located on the IncI1 plasmid. Primers linking the *bla*_CMY-2_-like gene to the IncI1 backbone [25] and/or S1 nuclease digestion/PFGE and hybridisation with IncI1 and *bla*_CMY-2_ probes (primers listed in S1 Table), as described previously [26], were used to confirm the genetic location of the *bla*_CMY-2_-like gene.

### Reference libraries for MLST

Existing *E*. *coli* MLST primers are located in conserved regions flanking a smaller gene fragment used as the MLST allele. As the whole amplicon is cleaved and analysed by MALDI-TOF MS, allele libraries must include the primer sequences and the ‘intervening regions’ between the primers and the MLST allele. Reference libraries for each MLST locus were compiled from the current list of allelic variants available from the *E*. *coli* MLST website (http://mlst.warwick.ac.uk/mlst/dbs/Ecoli/Downloads_HTML; accessed January 2015). A consensus ‘intervening’ sequence for each allele was generated from ~100 *E*. *coli* sequences available in GenBank, similar to the method described previously [10].

### MLST by MALDI-TOF MS

The seven target genes, *adk*, *fumC*, *gyrB*, *icd*, *mdh*, *purA* and *recA*, were amplified with primers based on published forward and reverse MLST primers (http://mlst.warwick.ac.uk/mlst/dbs/Ecoli/documents/primersColi_html) but with recognition sequences for either T7 (forward primer) or SP6 RNA polymerase (reverse primer) included (S1 Table). PCRs were performed in a final volume of 10 μl (per allele) with 0.02 U HotStarTaq® DNA polymerase (Qiagen, Doncaster, Australia), 1 x PCR buffer, 200 nM each dNTP, 200 nM of each of the forward and reverse primers and 2 μl of crude lysate as template (1:10 dilution). PCR conditions were: activation at 95°C for 15 min; 45 cycles of 95°C for 20 s, 60°C for 30 s, 72°C for 60 s; final extension at 72°C for three min. Amplicons were treated with shrimp alkaline phosphatase (SAP) (Agena Bioscience, San Diego, USA) to remove unincorporated dNTPs according to the manufacturer’s instructions.

Following SAP treatment, simultaneous reverse-transcription and base-specific cleavage reactions were performed according to the MassCLEAVE protocol (Agena Bioscience) [9]. In brief, each amplicon was subjected to four separate *in vitro* transcription and base-specific cleavage reactions, resulting in fragments cleaved in both forward and reverse directions at either T- (U-) or C- nucleotides [12]. Products of each reaction were desalted using anion-exchange resin and 8–12 nl dispensed onto a 96-well matrix-coated SpectroCHIP using the MassARRAY™ RS1000 Nanodispenser and analysed on a MassARRAY™ Analyzer 4 using SpectroAcquire 4.0 (Agena Bioscience). Resulting spectra were analysed using iSEQ software 1.0 (Agena Bioscience), which compares sample spectra to the spectral library generated from *in silico* cleavage of the allelic variants in the reference libraries. Allele numbers are assigned automatically based on the ‘best match’, calculated by an iterative process of analysing spectral features such as missing and additional peaks to produce a list of allelic variants ranked by ‘confidence score’ [9]. Samples with more than one best match (denoted with an asterisk by the iSEQ software) or with a confidence score ≤0.9 were manually reviewed (comparison of the top three highest ranked matches) for spectral features that supported assignment of a single best match. DNA sequencing was conducted if manual review was inconclusive. Allele combinations for each sample were entered into the MLST database for ST assignment. The repeatability of MLST by MALDI-TOF MS was evaluated using 10 of the isolates used for validation, representing diverse STs, repeated in triplicate.

### Discriminatory power of three versus seven alleles

Simpson’s index of diversity (*D*) is a measure of diversity in a population of *n* individuals and can be used as an indicator of the discriminatory power of different typing methods [27, 28]. We calculated *D* when all seven alleles were used and compared it to a three-allele approach, to determine whether the discriminatory power of MLST is affected when the number of alleles used to measure clonal diversity is reduced. The set of *E*. *coli* isolates carrying *bla*_CMY-2_ on an IncI1 plasmid was used to calculate *D* and confidence intervals (CI) were calculated as previously described [28].

## Results

### Evaluation of MLST by MALDI-TOF MS

Thirty-three *E*. *coli* isolates of known ST were used to evaluate the MALDI-TOF MS MLST method. MALDI-TOF MS assigned the expected allele number at all seven loci for 32/33 validation isolates and at six loci for the remaining isolate, corresponding to 230/231 correctly assigned alleles (99.6%). The single discrepant allele was assigned *icd*-306 instead of the expected *icd*-16. These two alleles differ by only one SNP and have almost identical spectral features (Table 1).

**Table 1:** Examples of discrepancies between MLST by MALDI-TOF MS and sequencing.

Allele	MALDI-TOF	Actual	Reason	Recommendation
*fumC*	273	24	Similar spectra/one SNP	Repeat or sequence^c^
*gyrB*	302	10	Similar spectra/one SNP^a^	Manual review for clarification
*gyrB*	304	4	Indistinguishable^b^	Sequence for clarification
*icd*	77	18	Similar spectra/one SNP	Repeat or sequence^c^
*icd*	306	16	Similar spectra/one SNP	Repeat or sequence^c^
*mdh*	290	7	Similar spectra/one SNP	Repeat or sequence^c^

^a^ These alleles differ by the presence/absence of a 10,290 Da peak that is often not detected by the software but is visible upon manual inspection. Other alleles that differ by the presence/absence of this peak are listed in S3 Table.

^b^ Indistinguishable alleles have identical spectral patterns for all four cleavage reactions. Other indistinguishable alleles can be determined by the simulation software.

^c^ Workflow for discrepant alleles detailed in S1 Fig.

### Repeatability

Ten isolates representing diverse STs were used to assess repeatability, but one allele from one sample failed MS repeatedly, generating a total of 69 alleles repeated in triplicate. Only four of the 69 alleles (two *fumC*, two *gyrB*) were not assigned the same number in all three replicates. In all cases, the software had assigned either multiple top matches or a single top match with a confidence score below 0.9, but manual review was unable to resolve a single best match. Repeatability was therefore calculated as 94.2% (65/69) overall, with discrepancies in *fumC* or *gyrB* only.

### Refinement of references libraries

In three samples *recA* was assigned *recA*-325 by MALDI-TOF MS but sequencing indicated that *recA*-2 was present. Manual inspection revealed that the reason for this discrepancy was a SNP in the left-hand intervening region that was not represented in the consensus sequence as it was present in only 20% of *E*. *coli* sequences analysed. The *recA* library was updated to include an additional *recA*-2 entry with the identified SNP in the intervening region and the three discrepant samples were reanalysed. All three *recA* alleles were then correctly identified as *recA*-2, highlighting the need for iterative improvement of the reference libraries. A similar update was made to the *fumC* reference library where an additional *fumC*-4 entry with a modified intervening region resulted in confident assignment of *fumC*-4 for two samples that were initially assigned multiple best matches (*fumC*-4 and -315). Two additional *mdh* entries (*mdh*-8 and -11) with modified intervening regions improved the power of the software to discriminate between *mdh*-8 and -283 and *mdh*-11 and -264, since these pairs of allele variants have nearly identical spectra.

### MLST of *E*. *coli* carrying IncI1*-bla*_CMY-2_ plasmids

PCR and/or S1 nuclease digestion/PFGE and Southern hybridisation were used to determine the genetic location of the *bla*_CMY-2_-like gene in 72 *E*. *coli* isolates identified as carrying both a *bla*_CMY-2_-like gene and an IncI1 plasmid in previous surveys [21, 24]. The *bla*_CMY-2_-like gene was confirmed to be located on the IncI1 plasmid in 62 of these isolates (S2 Fig). MLST by MALDI-TOF MS was used to investigate the clonality of these 62 isolates after refinements were made to the reference libraries, as discussed above. As a secondary validation, 10 isolates representing different STs to those in the initial validation set were also subjected to DNA sequencing as well as MLST by MALDI-TOF MS. The allele numbers obtained from sequencing were 100% concordant with those assigned by MALDI-TOF MS for all 10 isolates.

The remaining 52 isolates were subjected to MLST by MALDI-TOF MS only. Forty-five were assigned a known ST and seven were assigned a combination of alleles that did not correspond to a known ST, prompting manual inspection to confirm the assignment of each allele. Any alleles that were not confidently assigned the same number upon manual review were sequenced. Three of the seven isolates were subsequently assigned to a known ST after a single allele from each isolate was found to be misassigned by MALDI-TOF MS (one *fumC*, one *icd* and one *mdh*–Table 1). Two isolates were confirmed to have new STs (one with a new combination of alleles and one with a new *gyrB* variant) and two were found to be mixed at one or more loci, representing either heterogeneity at these loci or mixed samples containing more than one clone. These two mixed isolates were excluded from further analysis.

In total, 58/60 isolates were assigned to a known ST, corresponding to 34 different STs. Thirteen STs were represented more than once, but no ST was represented more than six times (Table 2). The most prevalent STC was STC38 (9/60) followed by STC10 and STC23 (both 5/60), and STC648 (3/60), but no single STC appeared to cluster temporally.

**Table 2:** Sequence types of *E*. *coli* strains carrying IncI1-*bla*_CMY-2_ plasmids.

ST^a^	STC^b^	n =	Year(s) isolated
10	10	2	2009,14
167	10	2	2007,14
617	10	1	2014
88	23	3	2008
410	23	2	2008,09
38	38	6	2008,09,13,14
963	38	3	2008,09,13
609	46	1	2013
69	69	1	2009
101	101	1	2008
131	131	2	2008,13
354	354	1	2006
405	405	1	2014
80	568	1	2014
648	648	2	2008,13
3384	648	1	2008
68	–	2	2009
120	–	1	2008
196	–	1	2014
224	–	1	2014
357	–	4	2008
457	–	5	2009,13,14
773	–	1	2005
973	–	1	2013
1049	–	1	2008
1161	–	1	2013
1193	–	2	2009
1303	–	1	2014
1588	–	1	2005
1598	–	1	2009
2178	–	1	2013
2541	–	2	2008,09
2787	–	1	2008
3498	–	1	2013
New^c^	–	2	2014

^a^ Sequence type

^b^ Sequence type complex based on http://mlst.warwick.ac.uk/mlst/dbs/Ecoli;–, no STC

^c^ One isolate has a new combination of alleles and one has a new *gyrB* variant.

### Discriminatory power of three versus seven alleles

Simpson’s index of diversity (*D*) was used to assess the utility of three alleles instead of seven to measure clonal diversity in the set of isolates carrying a *bla*_CMY-2_-like gene on an IncI1 plasmid (n = 60, excluding two mixed samples) [27, 28]. We selected *adk*, *fumC* and *recA* as they have previously been shown to be the most discriminatory alleles individually for determining ST [29] and the iSEQ simulation software can confidently differentiate the allelic variants of *adk*, *fumC* and *recA* (S2 Table). The discriminatory power of three alleles (*D* = 0.959, CI = 0.934–0.983) was not substantially different to that of all seven alleles (*D* = 0.975, CI = 0.960–0.989) (confidence intervals overlap) when used to measure the clonal diversity of isolates in this set.

## Discussion

Rapid and reproducible typing of *E*. *coli* is essential for effective infection control and for surveillance of known virulent and/or resistant subtypes. Here we showed that full *E*. *coli* MLST by MALDI-TOF MS is over 99% concordant with conventional MLST by DNA sequencing. Results are obtainable within 12 hours and the running costs for MALDI-TOF MS (approx. AUD$90/isolate) are comparable to bidirectional Sanger sequencing (approx. AUD$95/isolate) and currently lower than WGS (approx. AUD$155/isolate using Illumina MiSeq technology). While an initial investment in specialist equipment is required (approx. AUD$250,000), the MALDI-TOF MS has proven a valuable tool for a variety of diagnostic applications [13–15].

The utility of MLST by MALDI-TOF MS lies in its speed, high-throughput capacity and automated analysis that requires minimal operator expertise, enabling both large-scale population analyses and targeted screening for known pathogenic lineages in a diagnostic setting, with simple boiled lysates as the template. A simplified three-allele approach (*adk*, *fumC*, *recA*) would further reduce the cost (approx. AUD$40/isolate) and processing time per sample and in the population investigated here, the discriminatory power of three alleles was not substantially different to that of seven alleles, suggesting that this approach may suffice in some settings as a measure of population diversity.

The limitations of this method include a small error rate of less than 1% but errors can be detected if an isolate is assigned an unknown combination of alleles, prompting manual review and confirmatory sequencing. With iterative refinement of the reference libraries, including modification of the intervening consensus sequences and periodic updates to include new allelic variants, the error rate is expected to be progressively reduced. Furthermore, the incorrect allele number assignments that did occur were only one SNP different to the correct allele number and thus the sample was still assigned to the correct STC. Manual inspection was required for approximately 20% of alleles (either prompted by the software or with a confidence score of ≤0.9), but this process is relatively quick and by ensuring that adequate amounts of DNA (5 ng/ μl) are used in the cleavage reactions and 8–12 nl of cleaved product is dispensed onto the SpectroCHIP, the need for manual review may be reduced. Finally, new SNPs can be identified using this method [9] but must be confirmed by sequencing as new alleles can only be submitted to the MLST website if the isolate is sequenced by WGS methods.

Using MALDI-TOF MS MLST, we identified a high level of clonal diversity (*D* = 0.975) among *E*. *coli* strains from Sydney, Australia carrying a *bla*_CMY-2_-like gene on an IncI1 plasmid. Some STs identified here have been reported in association with *bla*_CMY-2_-like genes previously, such as ST131 and members of STC10 and STC38, and are associated with human disease [30–33], while other STs have been reported in isolates of animal or environmental origin (http://mlst.warwick.ac.uk/mlst/dbs/Ecoli/GetTableInfo_html). This pattern of polyclonality has been reported among *E*. *coli* carrying *bla*_CMY-2_ in other parts of the world [30, 31, 34] and reflects an interesting difference in the dynamics of spread compared with the more prominent *bla*_CTX-M-15_, which appears to have spread predominantly via its association with IncF plasmids in ST131 [35].

In conclusion, *E*. *coli* MLST by MALDI-TOF MS is a rapid alternative to DNA sequencing with minimal operator expertise required and when implemented in a clinical setting has the capacity to identify the spread of high-risk pathogenic lineages. We identified substantial clonal diversity among *E*. *coli* carrying *bla*_CMY-2_ on IncI1 plasmids from Sydney, Australia, implicating horizontal gene transfer rather than clonal expansion as an important dynamic in the dissemination of this resistance gene.

## Supporting Information

S1 Fig Workflow for the assignment of *Escherichia coli* sequence types by MALDI-TOF MS. (DOCX)

S2 Fig S1 nuclease digestion pulsed-field gel electrophoresis (PFGE) and Southern hybridisation. (DOCX)

S1 Table Primers used in this study. (DOCX)

S2 Table Simulation results for all seven *Escherichia coli* MLST alleles. (DOCX)

S3 Table *gyrB* allele pairs that differ by 10,290 Da peak. (DOCX)
