# Supplementary material for: MALDI-TOF Mass Spectrometry for Multilocus Sequence Typing of Escherichia coli Reveals Diversity among Isolates Carrying bla CMY-2-Like Genes
Source: PLoS One. 2015 Nov 20;10(11):e0143446. doi: 10.1371/journal.pone.0143446 (PMC4654469; doi:10.1371/journal.pone.0143446)
Supplement: S1 Fig — (DOCX) [file pone.0143446.s001.docx]

Allele number assignment

Confidence score >0.9?

YES

Known problem allele?

YES

NO

NO

Manually review top three matches

One best match?

YES

NO

Spectral quality poor?

YES

NO

Sequence

Repeat

**Upload**

Manual review

One best match?

YES

NO

Sequence

**Upload**

**Upload**

Known ST?

YES

NO

**REPORT**

Manually review conflicting allele/s

Any change to allele number assignment?

YES

NO

Sequence all targets to confirm new ST

**S1 Fig.** **Workflow for the assignment of *Escherichia coli* sequence types by MALDI-TOF MS.**

**Upload**

**Upload all seven alleles**
