# Supplementary material for: MALDI-TOF Mass Spectrometry for Multilocus Sequence Typing of Escherichia coli Reveals Diversity among Isolates Carrying bla CMY-2-Like Genes
Source: PLoS One. 2015 Nov 20;10(11):e0143446. doi: 10.1371/journal.pone.0143446 (PMC4654469; doi:10.1371/journal.pone.0143446)
Supplement: S2 Fig — (DOCX) [file pone.0143446.s002.docx]

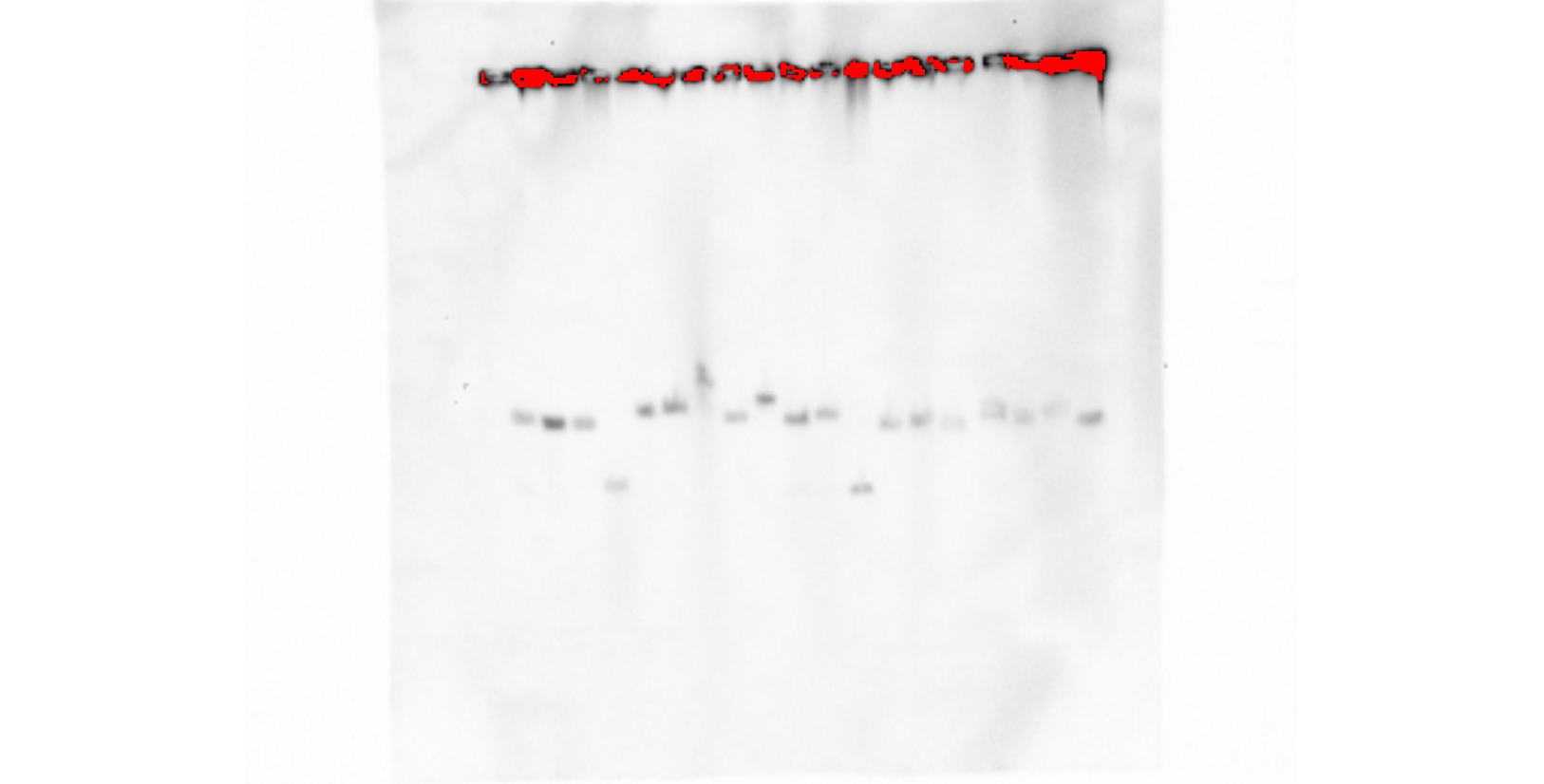

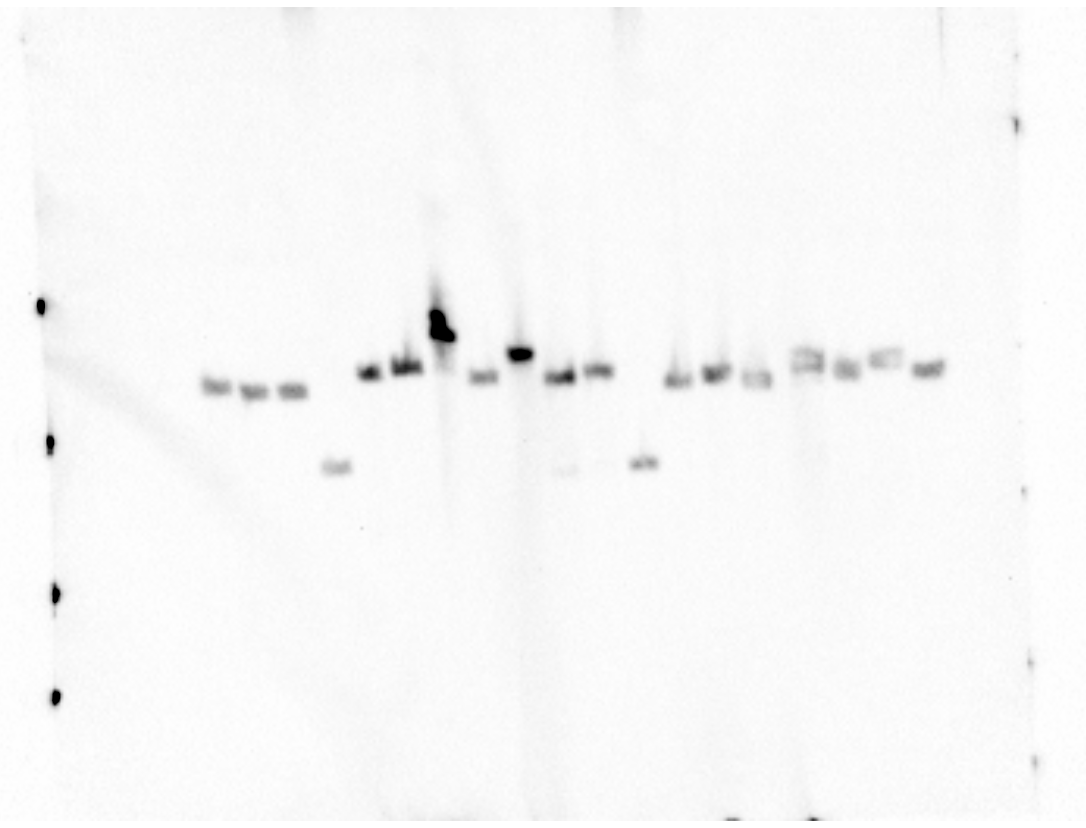

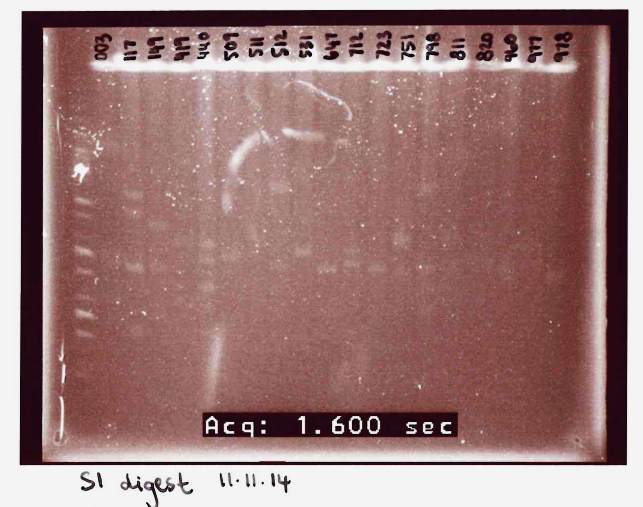


**1**

**2**

**3**

**4**

**5**

**6**

**7**

**8**

**9**

**10**

**11**

**12**

**1**

**2**

**3**

**4**

**5**

**6**

**7**

**8**

**9**

**10**

**11**

**12**

**1**

**2**

**3**

**4**

**5**

**6**

**7**

**8**

**9**

**10**

**11**

**12**

**48.5**

**63.5**

**82.0**

**145.5**

**97.0**

C

B

A

**S2 Fig. S1 nuclease digestion pulsed-field gel electrophoresis (PFGE) and Southern hybridisation.** (A) S1 nuclease digestion PFGE of 12 *E. coli* isolates and Southern hybridisation with *bla*_CMY-2_ (B) and IncI1 probes (C). Sizes (kb) of bands in the MidRange I PFG Marker (New England Biolabs, Ipswich, USA) are shown.
