# Supplementary material for: MALDI-TOF Mass Spectrometry for Multilocus Sequence Typing of Escherichia coli Reveals Diversity among Isolates Carrying bla CMY-2-Like Genes
Source: PLoS One. 2015 Nov 20;10(11):e0143446. doi: 10.1371/journal.pone.0143446 (PMC4654469; doi:10.1371/journal.pone.0143446)
Supplement: S1 Table — (DOCX) [file pone.0143446.s003.docx]

**Table S1.** **Primers used in this study.**

| Primer name | Sequence^a^ | Reference |
| --- | --- | --- |
| CITMF | TGGCCAGAACTGACAGGCAAA | [[1](#_ENREF_1)] |
| CITMR | TTTCTCCTGAACGTGGCTGGC | [[1](#_ENREF_1)] |
| I1 F | CGAAAGCCGGACGGCAGAA | [[2](#_ENREF_2)] |
| I1 R | TCGTCGTTCCGCCAAGTTCGT | [[2](#_ENREF_2)] |
| IncI1-1 | AGTTCATCTCTGCCTTTGT | [[3](#_ENREF_3)] |
| colIa-R | CGCTCACAACAATAGAATG | [[3](#_ENREF_3)] |
| iseq_adkF | cagtaatacgactcactatagggagaaggctATTCTGCTTGGCGCTCCGGG | This study |
| iseq_adkR | cgatttaggtgacactatagaagagaggctCCGTCAACTTTCGCGTATTT | This study |
| iseq_fumCF | cagtaatacgactcactatagggagaaggctTCACAGGTCGCCAGCGCTTC | This study |
| iseq_fumCR1 | cgatttaggtgacactatagaagagaggctTCCCGGCAGATAAGCTGTGG | This study |
| iseq_gyrBF | cagtaatacgactcactatagggagaaggctTCGGCGACACGGATGACGGC | This study |
| iseq_gyrBR1 | cgatttaggtgacactatagaagagaggctGTCCATGTAGGCGTTCAGGG | This study |
| iseq_icdF | cagtaatacgactcactatagggagaaggctATGGAAAGTAAAGTAGTTGTTCCGGCACA | This study |
| iseq_icdR | cgatttaggtgacactatagaagagaggctGGACGCAGCAGGATCTGTT | This study |
| iseq_mdhF1 | cagtaatacgactcactatagggagaaggctAGCGCGTTCTGTTCAAATGC | This study |
| iseq_mdhR1 | cgatttaggtgacactatagaagagaggctCAGGTTCAGAACTCTCTCTGT | This study |
| iseq_purAF | cagtaatacgactcactatagggagaaggctCGCGCTGATGAAAGAGATGA | This study |
| iseq_purAR | cgatttaggtgacactatagaagagaggctCATACGGTAAGCCACGCAGA | This study |
| iseq_recAF1 | cagtaatacgactcactatagggagaaggctACCTTTGTAGCTGTACCACG | This study |
| iseq_recAR1 | cgatttaggtgacactatagaagagaggctAGCGTGAAGGTAAAACCTGTG | This study |

^a^ T7 (forward) and SP6 (reverse) recognition sequences are shown in lower case. Published MLST primer sequences (<http://mlst.warwick.ac.uk/mlst/dbs/Ecoli/documents/primersColi_html>) are shown in upper case.

**References**

1. Pérez-Pérez FJ, Hanson ND. Detection of plasmid-mediated AmpC β-lactamase genes in clinical isolates by using multiplex PCR. J Clin Microbiol. 2002;40:2153-62.

2. Carattoli A, Bertini A, Villa L, Falbo V, Hopkins KL, Threlfall EJ. Identification of plasmids by PCR-based replicon typing. J Microbiol Methods. 2005;63:219-28.

3. Tagg KA, Iredell JR, Partridge SR. Complete sequencing of IncI1 sequence type 2 plasmid pJIE512b indicates mobilization of *bla*_CMY-2_ from an IncA/C plasmid. Antimicrob Agents Chemother. 2014;58:4949-52.
