# Supplementary material for: MALDI-TOF Mass Spectrometry for Multilocus Sequence Typing of Escherichia coli Reveals Diversity among Isolates Carrying bla CMY-2-Like Genes
Source: PLoS One. 2015 Nov 20;10(11):e0143446. doi: 10.1371/journal.pone.0143446 (PMC4654469; doi:10.1371/journal.pone.0143446)
Supplement: S2 Table — (DOCX) [file pone.0143446.s004.docx]

**S2 Table.** **Simulation results for all seven *Escherichia coli* MLST alleles.**

| Allele | High power^a^ | Medium-low power^b^ |
| --- | --- | --- |
| *adk* | 99.67 | 0.33 |
| *fumC* | 97.25 | 2.75 |
| *gyrB* | 96.75 | 3.25 |
| *icd* | 99.78 | 0.22 |
| *mdh* | 99.70 | 0.30 |
| *purA* | 96.74 | 3.26 |
| *recA* | 100 | 0 |

^a^ Percentage of alleles differentiated by >8 spectral features (missing peaks, additional peaks, peak intensity differences) using all four cleavage reactions.

^b^ Percentage of alleles differentiated by 2-8 spectral features using all four cleavage reactions.
