# Supplementary material for: MALDI-TOF Mass Spectrometry for Multilocus Sequence Typing of Escherichia coli Reveals Diversity among Isolates Carrying bla CMY-2-Like Genes
Source: PLoS One. 2015 Nov 20;10(11):e0143446. doi: 10.1371/journal.pone.0143446 (PMC4654469; doi:10.1371/journal.pone.0143446)
Supplement: S3 Table — (DOCX) [file pone.0143446.s005.docx]

**S3 Table.** ***gyrB* allele pairs that differ by 10,290 Da peak.**

| Allele pair | Nucleotide change^a^ |
| --- | --- |
| 4/194 | C/T |
| 22/270 | C/T |
| 32/14 | C/T |
| 69/347 | C/T |
| 85/296 | C/T |
| 101/70 | C/T |
| 109/1 | C/T |
| 221/53 | C/T |
| 266/64 | C/T |
| 10/302 | no insertion/9-bp insertion^b^ |

^a^ A nucleotide change at position 288 in the *gyrB* allele corresponds to the presence/absence of the 10,290 Da peak.

^b^ Insertion of 9 bases (CGACAAGCG) at position 288 also corresponds to the presence/absence of the 10,290 Da peak.
